# Dental Anatomy

**Published:** 1890-05

**Authors:** 


### Dental Anatomy.

A Manual of Dental Anatomy, Human and Comparative, by Charles S. Tomes, M.A., F.R.S. 212 Illustrations. Third Edition.

This work makes its appearance from the publication house of P. Blakiston, Sons & Co., Philadelphia. In the preface to this edition the author says, "It has been my endeavor to give an outline of the more important facts of odontology, such as may serve for an introduction to a more extended study of the subject. Within the limitations of an elementary text book more than this seems not to be practicable. I have therefore omitted, or passed by with brief mention, hypothesis as to the genesis of the teeth and the like."

"The rule which I have followed has been to give references only to works and papers, which might be regarded, as in a measure, classical, and to those which, by reason of being somewhat new, have not yet found their way into systematic treatises."

This edition has been very carefully prepared and made more complete, in some respects, than either of the preceding editions. It has become a standard work, one that is, or ought to be used by all students in dental science. It is also valuable to the practitioner as a book of reference.

Mr. Tomes' fitness and ability for the preparation to such a work is fully admitted everywhere. His training from the beginning of his dental pupilage to the present time has been such as to fit him in an eminent degree for the preparation of a work of this kind. He has both natural and acquired endowments, which eminently fit him for the study and presentation of the subject of dental anatomy. The literary character of the work is unexceptionable, and is such as to be readily understood by the student.

The illustrations are very good indeed; so drawn and executed as to give a correct idea of that which is represented.

The dental profession owes Mr. Tomes a debt of gratitude, and more, which will never be fully discharged. The best way, however, to do this is for every one to become an earnest student of the subject as presented in this work. The work can be procured through any bookseller.

